# Population genomics reveals additive and replacing horizontal gene transfers in the emerging pathogen *Dickeya solani*

**DOI:** 10.1186/s12864-015-1997-z

**Published:** 2015-10-14

**Authors:** Slimane Khayi, Pauline Blin, Jacques Pédron, Teik-Min Chong, Kok-Gan Chan, Mohieddine Moumni, Valérie Hélias, Frédérique Van Gijsegem, Denis Faure

**Affiliations:** Institute for Integrative Biology of the Cell (I2BC), CNRS CEA Univ. Paris-Sud, Université Paris-Saclay, Saclay Plant Sciences, Avenue de la Terrasse, 91198 Gif-sur-Yvette cedex, France; Université Moulay Ismaïl, Faculté des Sciences, Département de Biologie, Meknès, Morocco; UPMC Univ Paris 06, UMR 7618, IEES Paris (Institute of Ecology and Environmental Sciences), 7 Quai Saint bernard, 75005 Paris, France; Division of Genetics and Molecular Biology, Institute of Biological Sciences, Faculty of Science, University of Malaya, 50603 Kuala Lumpur, Malaysia; Fédération Nationale des Producteurs de Plants de Pomme de Terre-Recherche développement Promotion du Plant de Pomme de Terre (FN3PT-RD3PT), 75008 Paris, France; UMR 1349 IGEPP INRA - Agrocampus Ouest Rennes, 35653 LeRheu, France; INRA, UMR 1392, IEES Paris (Institute of Ecology and Environmental Sciences), 7 Quai Saint Bernard, 75005 Paris, France

**Keywords:** Dickeya, Soft rot, Potato, Population genomics, Horizontal gene transfer

## Abstract

**Background:**

*Dickeya solani* is an emerging pathogen that causes soft rot and blackleg diseases in several crops including *Solanum tuberosum*, but little is known about its genomic diversity and evolution.

**Results:**

We combined Illumina and PacBio technologies to complete the genome sequence of *D. solani* strain 3337 that was used as a reference to compare with 19 other genomes (including that of the type strain IPO2222^T^) which were generated by Illumina technology. This population genomic analysis highlighted an unexpected variability among *D. solani* isolates since it led to the characterization of two distinct sub-groups within the *D. solani* species. This approach also revealed different types of variations such as scattered SNP/InDel variations as well as replacing and additive horizontal gene transfers (HGT). Infra-species (between the two *D. solani* sub-groups) and inter-species (between *D. solani* and *D. dianthicola*) replacing HGTs were observed. Finally, this work pointed that genetic and functional variation in the motility trait could contribute to aggressiveness variability in *D. solani*.

**Conclusions:**

This work revealed that *D. solani* genomic variability may be caused by SNPs/InDels as well as replacing and additive HGT events, including plasmid acquisition; hence the *D. solani* genomes are more dynamic than that were previously proposed. This work alerts on precautions in molecular diagnosis of this emerging pathogen.

**Electronic supplementary material:**

The online version of this article (doi:10.1186/s12864-015-1997-z) contains supplementary material, which is available to authorized users.

## Background

Pectinolytic bacteria belonging to the *Dickeya* and *Pectobacterium* genera are pathogens that cause soft rot and blackleg diseases in a wide range of plants and crops including *Solanum tuberosum* [1, 2]. These phytopathogens produce plant cell-wall degrading enzymes that are able to macerate the tuber and stem tissues, thus provoking the disease symptoms [3]. Since 2000s, the emerging *D. solani* species has been proposed as a contributor to the increased incidence of blackleg and soft rot diseases on potato crop in Europe and the Mediterranean basin [4]. The *D. solani* species has been officially described recently [5].

Little is known about the ecological and genetic traits that may support the relative success of *D. solani* in invading potato fields [6, 7]. *D. solani* can initiate disease from a low inoculum level in warm climates and was described in some studies to spread more easily through vascular tissues than other *Dickeya* species [4, 8]. Besides classical intergenic spacers 16S-23S rDNA [9], several molecular studies have proposed different marker genes for the identification of *D. solani* strains collected from potato and ornamental plants, such as *dnaX* [10], *recA* [11] and *fliC* [12]. At whole genome level, genomic and metabolic comparisons of two *D. solani* strains Ds0432-1 (isolated in Finland) and 3337 (isolated in France) vs. *D. dadantii* 3937 indicated a conserved synteny between the two species, but also the presence of distinctive traits [13, 14]. *D. solani* and *D. dadantii* diverged in their battery of non-ribosomal peptide/polyketide synthase clusters, T5SS/T6SS-related toxin-antitoxin systems and several metabolic abilities. Some of these traits would contribute to the successful invasion of this pathogen [13, 14]. More recently, a reverse genetic approach revealed that the virulence master regulators are quite the same in *D. solani* and *D. dadantii* [7].

The analysis of population genome structure and dynamics, including additive or replacing horizontal gene transfer (HGT) may bring valuable clues on the mechanisms of emergence of *D. solani*. While additive HGT allows the acquisition of novel genes by a population [15–20], replacing HGT provokes the replacement of an allele by another from close relatives [21]. HGT events inform about the genome diversification and adaptation processes, but also on the companion populations that the pathogens met during the emergence and dissemination steps. Replacing HGT is also of a major stake in pathogen diagnostic, as it may provoke false identification when the alleles exchanged by replacing HGT are used as molecular taxonomic markers.

Here, we analyzed the whole genome polymorphism of 20 *D.* solani isolates, including the type strain IPO2222^T^, collected from different geographic locations, dates of isolation and plant hosts. We combined Illumina and PacBio technologies to complete the 3337 *D. solani* strain genome that we used as a reference in the comparative genomics. While most strains belonged to a core-population that exhibited less than one hundred variant positions between two given genomes, some other genomes revealed massive replacing HGT from the companion pathogen *D. dianthicola* and a plasmid acquisition from *Burkholderia ambifaria*. Moreover, we were able to correlate SNPs in virulence genes with a decrease in aggressiveness, highlighting the power of genomics as a tool to reveal functional variability in *D. solani* population. To our knowledge this is the first study that reports whole genome analysis of a *D. solani* population and describes its diversity.

## Results

### *Complete genome of the* D. solani *3337*

The *D. solani* 3337 genome was previously sequenced by Illumina technology using two libraries (mate-pair and paired-end) and *de novo* assembled in a high quality draft genome deposited at NCBI [22]. In this work, the 3337 *D. solani* genome was re-sequenced using PacBio technology. The PacBio sequencing generated six contigs (2 473 62 pb, 1 512 701 bp, 894 591 bp, 49 337 bp, 10 627 bp, and 4 290 bp) with an average 150 fold coverage. The published Illumina-scaffolding was confirmed and the remaining gaps were filled using the PacBio contigs. Hence, combining the Illumina and PacBio sets of sequences, we obtained a complete sequence of the unique circular chromosome (4 922 460 bp). The RAST annotation generated 4 536 CDS and 97 RNAs. The *D. solani* 3337 complete genome was used as a reference for comparative genomics.

### *Positioning the sequenced* D. solani *strains within the* Dickeya *genus*

**Fig. 1:**
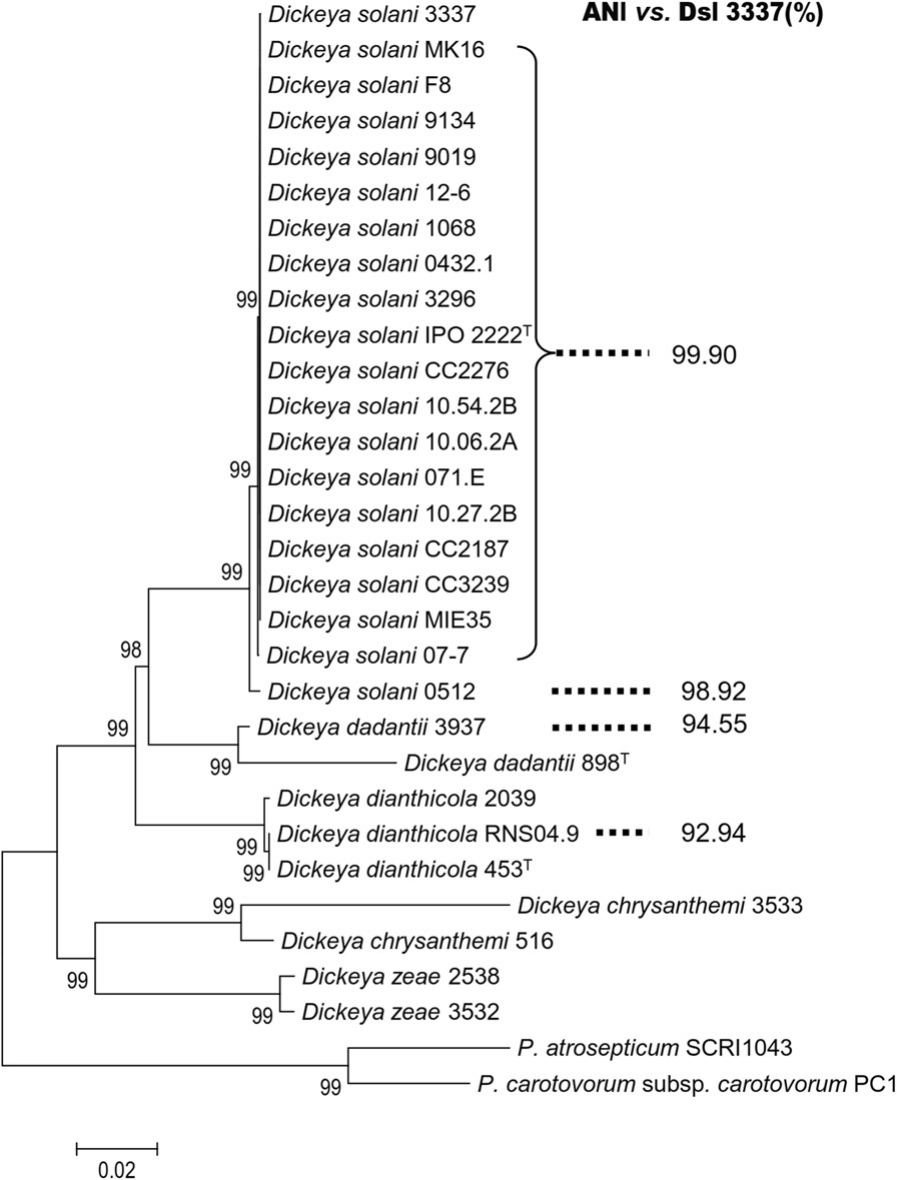
MLSA and ANIs of *D. solani* strains. In MLSA the sequences of the genes (*rpoD, gyrB, recA, rpoS, dnaX, dnaA, gapA, fusA, rplB, purA, gyrA*) were aligned with ClustalW, and a Neighbour-joining tree was created by Bootstrap method with 1000 bootstrap replications. ANI values were calculated using Dsl 3337 as a reference

In addition to *D. solani* 3337, 19 *D. solani* strains including the type strain IPO2222^T^ were collected at different years and geographical locations (Additional file 1: Table S1) and their genomes sequenced by Illumina technology. All these draft and complete genomes were used in multi-locus sequence analysis (MLSA) and average nucleotide identity (ANI) calculation. For MLSA, the eleven concatenated *rpoD, gyrB, recA, rpoS, dnaX, dnaA, gapA, fusA, rplB, purA, gyrA* housekeeping genes (17 298 bp) were aligned to construct a relation-tree using Neighbor-Joining method, the evolutionary distances were computed using the Maximum Composite Likelihood method [23]. All the *D. solani* (Dsl) isolates were grouped in a same cluster that was separated from the other pectinolytic enterobacteria (Fig. 1). Noticeably, the strain Dsl 0512 was the unique strain that was consistently distant from the other *D. solani* strains. As previously reported [5], within the genus *Dickeya* the most related species to *D. solani* were *D. dadantii* and *D. dianthicola*. ANI values which were calculated using the strain Dsl 3337 as a reference were in accordance with the MLSA clustering. All the *D. solani* strains exhibited an ANI value equal to or above 99.9 %, but that of Dsl 0512 was below 99 %. Among strains of the closest species, *D. dadantii* and *D. dianthicola* ANI values dropped to 94 % and 92 %, respectively.

### *Overview of the SNP and InDel variations* in D. solani *genomes*

Illumina reads of the *D. solani* strains were mapped on the complete genome sequence of Dsl 3337. The percentage of mapped reads was above 99 % for all strains with the exception of Dsl 9019 (98.08 %) and 0512 (92.34 %) (Additional file 1: Table S2). The mapping vs. Dsl 3337, which reached a high mean coverage value (between 400 and 900), allowed us to identify variations (SNPs and InDels) in each of the genomes (Additional file 1: Table S3). According to the number of variations, the *D. solani* strains could be clustered into three groups. The first group, which we thereafter term as the core-population, encompassed most of the strains (including IPO2222^T^ and the reference Dsl 3337) with a variation number ranging from 43 to 85. In the second group were the strains Dsl 07-7, 9019 and 9134 with a variation number between 1454 and 3433. The third group consisted in the only strain Dsl 0512 with a very high variation number that reached 37493. RAST annotation of the strain Dsl 3337 was used to position the variations in or out coding DNA sequences (CDSs), as well as to identify non-synonymous variations in CDSs (Additional file 1: Table S3). Non-synonymous variations ranged between 14 and 21 % of the total number of variations, hence only 6 to 18 non-synonymous variations were identified in strains of the *D. solani* core-population (Additional file 1: Table S3).

### *Heterogeneous distribution of the SNP and InDel variations* in D. solani *genes*

**Fig. 2:**
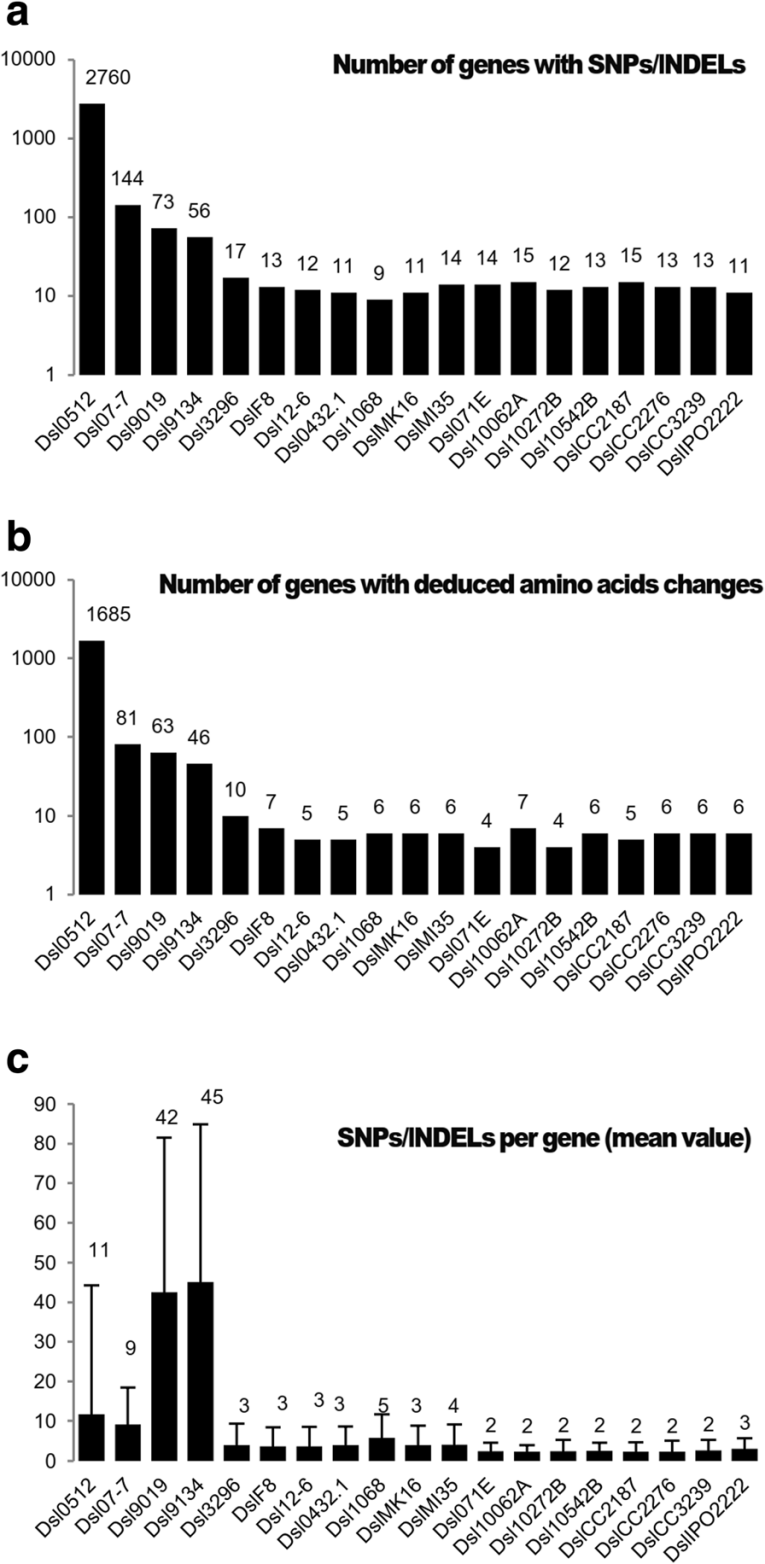
Number of genes affected by variations (SNPs and InDels). **a** Total number of genes affected by the variations for each strain. **b** Number of genes revealing amino acids change further variation. **c** The average number of variations affecting the genes in each strain

We refined our analysis by calculating the number of genes (CDSs) that were affected by SNPs and InDels as well as non-synonymous variations (Fig. 2a-b). In the core-population, 9 to 17 genes exhibited variations and about one half of them (4 to 10) harbored non-synonymous variations. In Dsl 07-7, 9019 and 9134, 56 to 144 genes were affected; and among them, 46 to 81 contained non-synonymous variations. In Dsl 0512, 2760 genes, hence half of the genome showed variations. To compare variation abundance in genes, a mean value of the number of all variations (synonymous and non-synonymous) per affected gene was calculated (Fig. 2c). In the core-population, this value ranged from 2 to 5. In Dsl 0512 and 07–7, it was similar (11 and 9, respectively) while the highest value was observed in Dsl 9134 and 9019 (45 and 42, respectively). Overall, these analyses revealed that Dsl 0512, 07–7, 9134 and 9019 harbored genes with different numbers of variations as compared to those in the core-population, suggesting putative HGTs from distinct sources.

**Fig. 3:**
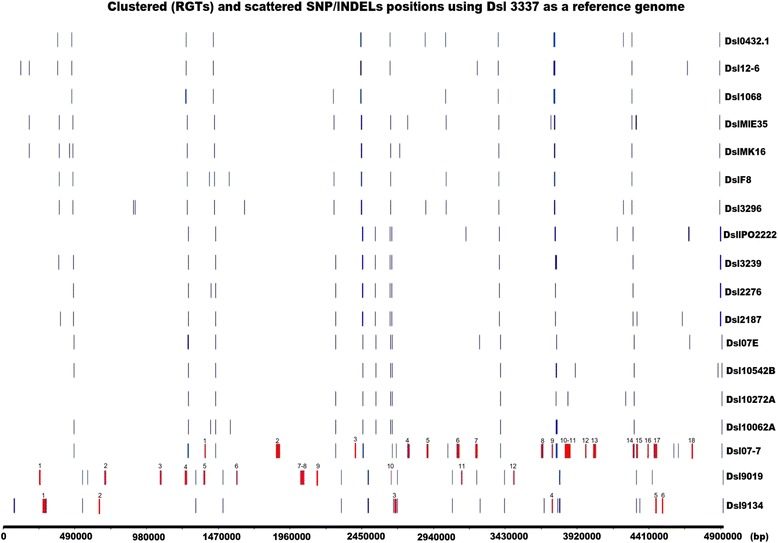
Mapping of the clustered and scaterred SNP/InDel variations using Dsl 3337 as a reference genome. Small colored sticks indicate variations positions: the scattered SNP/InDels are in blue color, while the clustered SNP/InDels (RGTs) are in red color and are numbered according to their successive position along the chromosome. Dsl 0512 is excluded from this figure due to high number of variations

Finally, all these different variations were positioned along the Dsl chromosome (Fig. 3). In the core-population, the rare variations appeared to be scattered with a mean distribution of 0.015 variations per kbp (when all SNPs and InDels were counted) and 0.012 variations per gene (when only SNPs and InDels in CDSs were counted). In Dsl 9134, 9019 and 07–7, most of the variations affected several tens of genes that are clustered in distinct regions, while only a few variations remain scattered. In Dsl 0512, variations exhibited a genome wide distribution. In the next part of the work, the three types of SNP/InDel distribution (scattered, clustered and wide genome distribution) have been analyzed in details.

### *The mosaic genome of* D. solani *0512 might define a novel* D. solani *sub-group*

Dsl 0512 differed from the other *D. solani* by the high number and wide distribution of variations (Additional file 1: Table S3, Figs. 2 and 3), a unique phylogenetic position in MLSA (Fig. 1), and a high percentage (7.66 %) of unmapped reads against Dsl 3337 genome (Additional file 1: Table S2). Unmapped reads were used for a *de novo* assembly which generated six contigs with a size ranging from 13 248 bp to 36 630 bp. All these six contigs were absent from the other *D. solani* strains. Using MAUVE [24], these six sequences were positioned on the draft genome of Dsl 0512 that was constructed using the strain Dsl 3337 as a reference (Additional file 2: Figure S1). RAST annotation indicated that most of the genes belonging to these 6 contigs coded for phage elements and hypothetical or unknown proteins, with the exception of some genes coding for two putative ABC transporters, two putative virulence factors and one methyl-accepting chemotaxis protein, all being carried by the contig4. The similarity scores were too weak to assign a more precise function and phylogenetic origin to these putative genes/proteins.

**Fig. 4:**
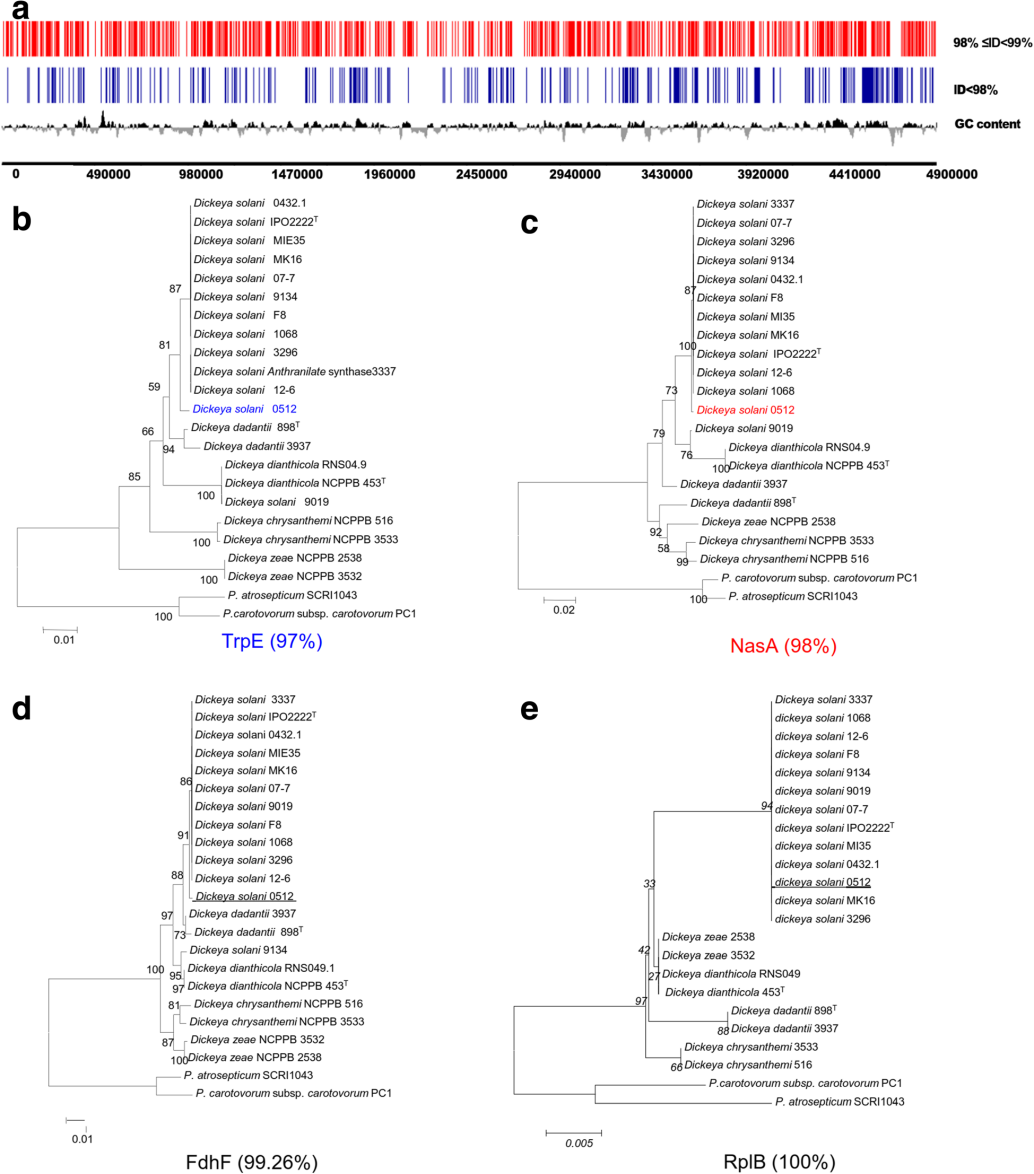
Mapping and phylogeny of the Dsl 0512 variant genes. In panel **a**, Position of variant genes of Dsl 0512 on the reference strains Dsl 3337. The panels **b**, **c**, **d** and **e** exemplify phylogenetic trees of selected proteins with different percentage of nucleotide identity

Another characteristic of Dsl 0512 was a high number of genes (half of the genome) that exhibited variations. These genes were distributed along the genome without any clustering in specific regions. Constructed phylogenetic trees revealed that the analyzed genes exhibiting a nucleotide identity below 98 % (compared to Dsl 3337 genes) did not belong to the core population gene cluster (Fig. 4, Additional file 3: Figure S2, Additional file 4: Figure S3, Additional file 5: Figure S4, Additional file 6: Figure S5). All these features supported the existence of a novel *D. solani* sub-group. The strain Dsl 0512 could be proposed as the eponym of the *D. solani* 0512 sub-group.

### Infra-species replacing HGT in Dsl 07-7

**Fig. 5:**
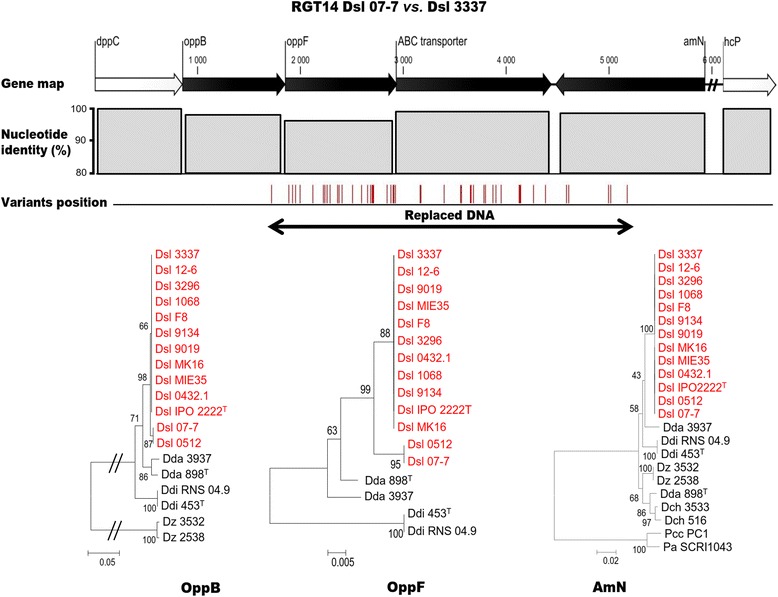
Replacing HGT region 14 (RGT14_07-7_) in *D .solani* 07-7. Gene map indicates the synteny conservation with Dsl 3337. The nucleotide identity decreases and the variation number increases at the positions of DNA acquisition, hence affecting the phylogenetic relationship of the encoded proteins

The 144 variant genes of Dsl 07-7 showed a non-uniform distribution along the chromosome, since most of them were clustered in 18 separate regions. One of these regions, which is presented in Fig. 5, contained four genes: *oppB*, *oppF*, an ABC transporter gene and *amN*. These genes contained in total 47 variations leading to a decrease of their nucleotide identity as compared to the corresponding genes in the Dsl 3337 genome. Moreover, the phylogenetic analysis of the protein sequence coded by the *oppB* and *oppF* genes, which were the most affected by variations, revealed a replacing HGT from a strain belonging to the *D. solani* 0512 sub-group. The 17 other regions exhibited a similar gene organization and a phylogenetic clustering with Dsl 0512 genes. Hence, all these 18 regions were called as RGT (replacing HGT) regions. They were numbered according to their successive position along the chromosome with the strain name in subscript position: RGT1_07-7_, RGT2_07-7_, RGT3_07-7_ … (Fig. 3). This analysis suggested that Dsl 07-7 acquired a dozen of gene fragments during massive replacing HGT(s) from strain(s) belonging to the Dsl 0512 sub-group. Hence, Dsl 07-7 exemplified the occurrence of an infra-specific gene exchange among the *D. solani* population, and also supported the possible co-existence of strains of the *D. solani* 0512 sub-group with those of the core-population.

### *Inter-species replacing HGT in* D. solani *strains 9134 and 9019*

In Dsl 9134, 39 among the 56 genes with variations were clustered in 6 RGT regions, the other genes with variations being scattered along the chromosome. In Dsl 9019, 63 among the 73 genes with variations were clustered in 12 RGT regions. In both strains, the RGT regions were named according to the same nomenclature as in Dsl 07-7 (Fig. 3).

**Fig. 6:**
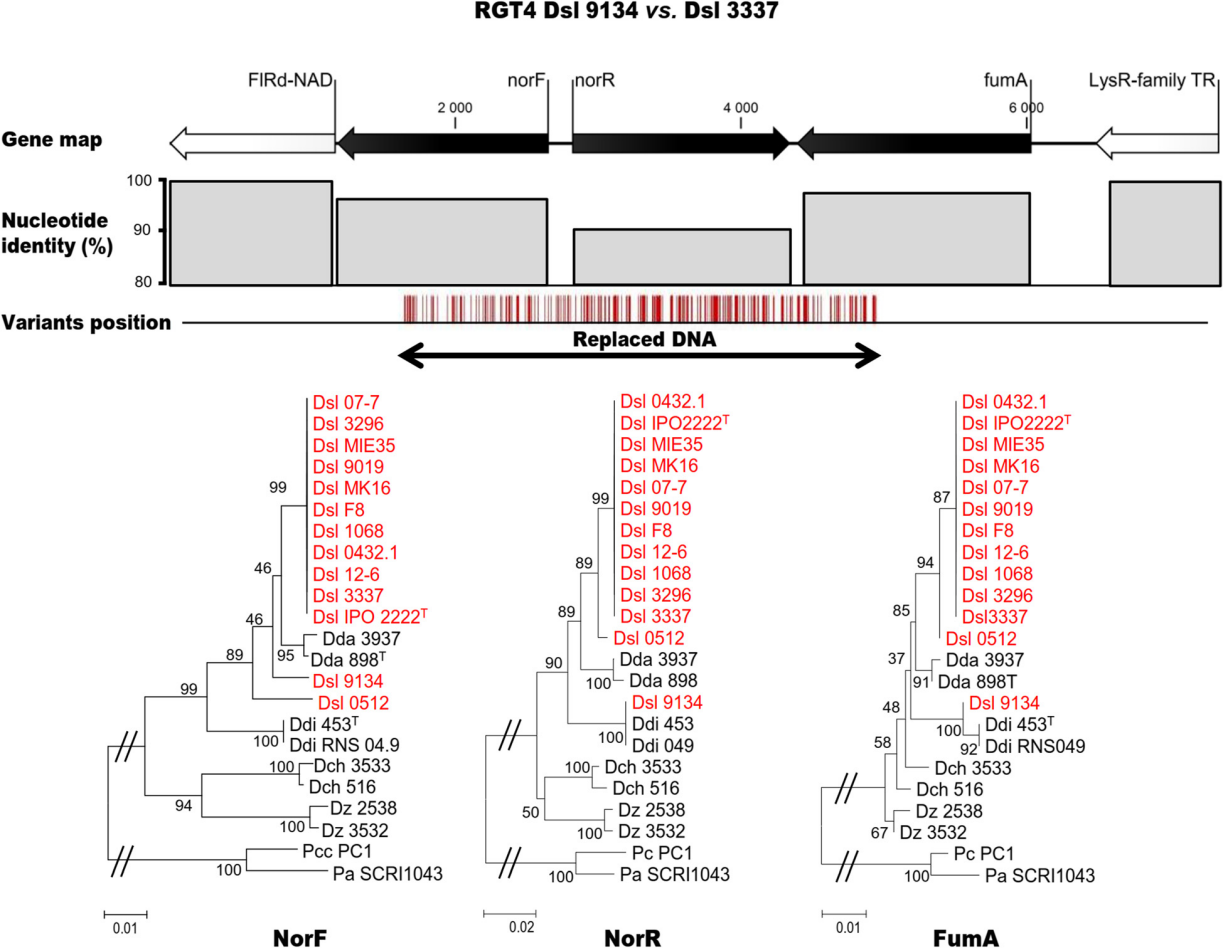
Replacing HGT region 4 (RGT4_9134_) in *D. solani* 9134. Gene map indicates the synteny conservation with Dsl 3337. The nucleotide identity decreases and the variation number increases at the position of DNA acquisition, hence affecting the phylogenetic relationship of the encoded proteins

**Fig. 7:**
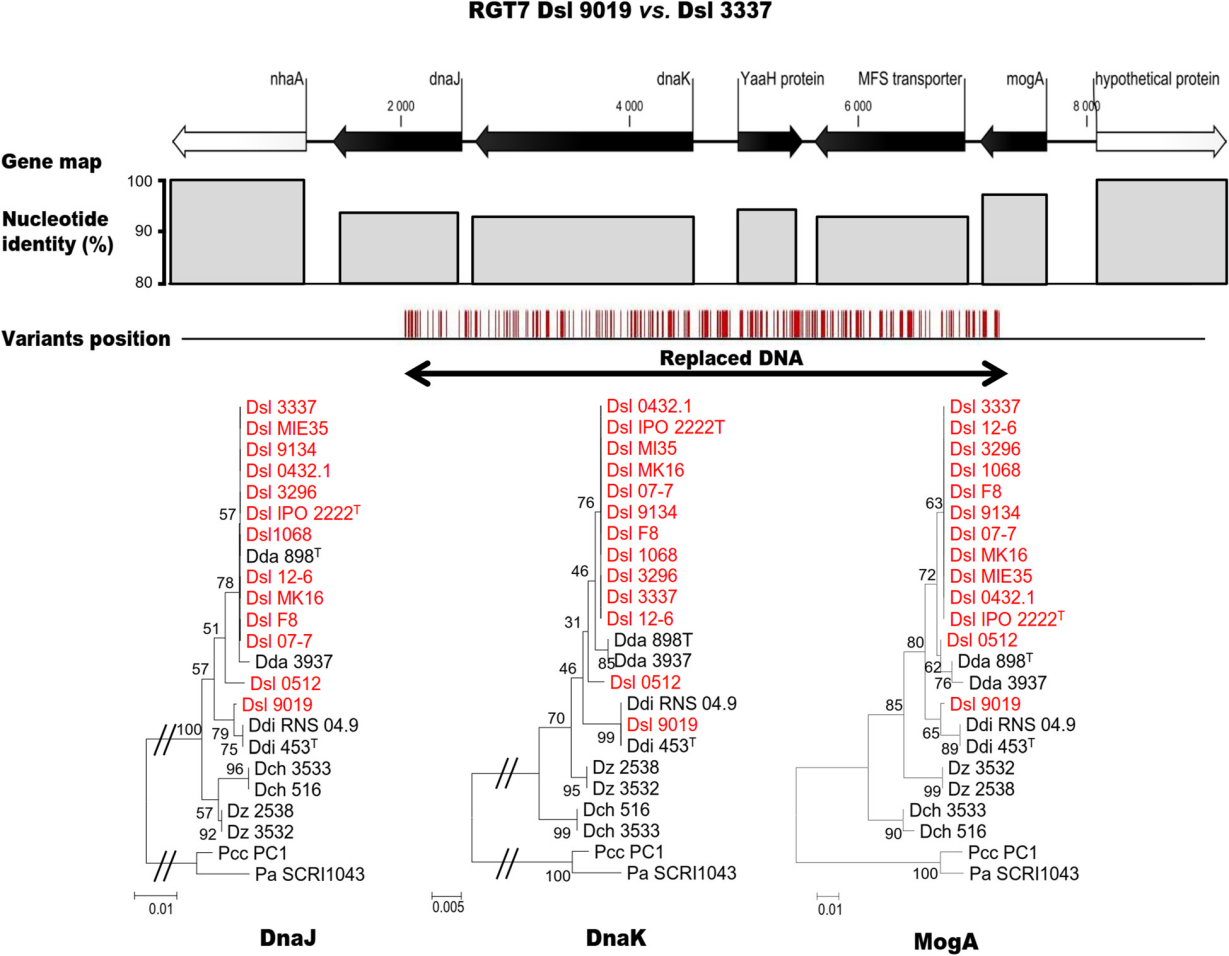
Replacing HGT region 7 (RGT7_9019_) in *D. solani* 9019. Gene map indicates the synteny conservation with Dsl 3337. The nucleotide identity decreases and the variant number increase at the position of DNA acquisition, hence affecting the phylogenetic relationship of the encoded proteins

The RGT4_9134_ illustrated the typical organization of these RGTs in Dsl 9134 (Fig. 6). RGT4_9134_ (4860 bp) exhibited 229 positions of variations that were distributed in three genes: *norF*, *norR*, and *fumA.* These genes were related to the nitric oxide metabolism. Because of the high number of variations, the gene identity with *D. solani* strain 3337 decreased in RGT4_9134_, especially in the *norR* gene that was located in the central part of the RGT region. Protein phylogeny revealed that the three proteins encoded by the RGT4_9134_ genes did not branch with their *D. solani* counterparts but were most closely related to those of *D. dianthicola*. The variation positions suggested that replacing HGT occurred in the middle of the genes *norF* and *fumA*, and hence generated proteins with an intermediate position between the *D. solani* and *D. dianthicola* proteins in the phylogenetic trees. A second example of inter-species replacing HGT is given with the RGT7_9019_ (6248 bp) of Dsl 9019, which contained five genes *dnaJ*, *dnaK*, *yaaH*, a MFS transporter gene and *mogA* (Fig. 7). This example highlighted that replacing HGT might also affect genes such as *dnaK* and *dnaJ* which are used for MLSA and taxonomic identification [25]. In RGT7_9019_, 269 variants were detected. Discrepancies within DnaJ, DnaK and MogA phylogenies suggested the occurrence of a replacing HGT from *D. dianthicola*. In all the other RGTs of Dsl 9134 and 9019, a phylogeny approach (Additional file 5: Figure S4, Additional file 6: Figure S5) also supported the occurrence of a replacing HGT using *D. dianthicola* population as the unique source.

### *Plasmid acquisition in* D. solani *strain 9019 from* Burkholderia

In addition to replacing HGT, an additive HGT event that consisted in a plasmid acquisition occurred in Dsl 9019. The Dsl 9019 unmapped reads, which represented 1.9 % of the total read number (Additional file 1: Table S2), allowed the generation of a single contig (43 564 bp) by *de novo* assembly. This plasmid exhibited a complete identity (100 %) with a plasmid of *Bulkholderia ambifaria* AMMD (CP000443.1). The stable replication of this plasmid in Dsl 9019 was verified in sub-cultures using plasmid-specific primers (pF1: cagcgaagagcaagacaa, pR1: tcatggaagcgatctcgg and pF2: ttaccggacgccgagctgtggcgt, pR2 :caggaagatgtcgttatcgcgagt).

### *In* D. solani *3296, variations in flagellar genes correlated motility and virulence decrease*

**Fig. 8:**
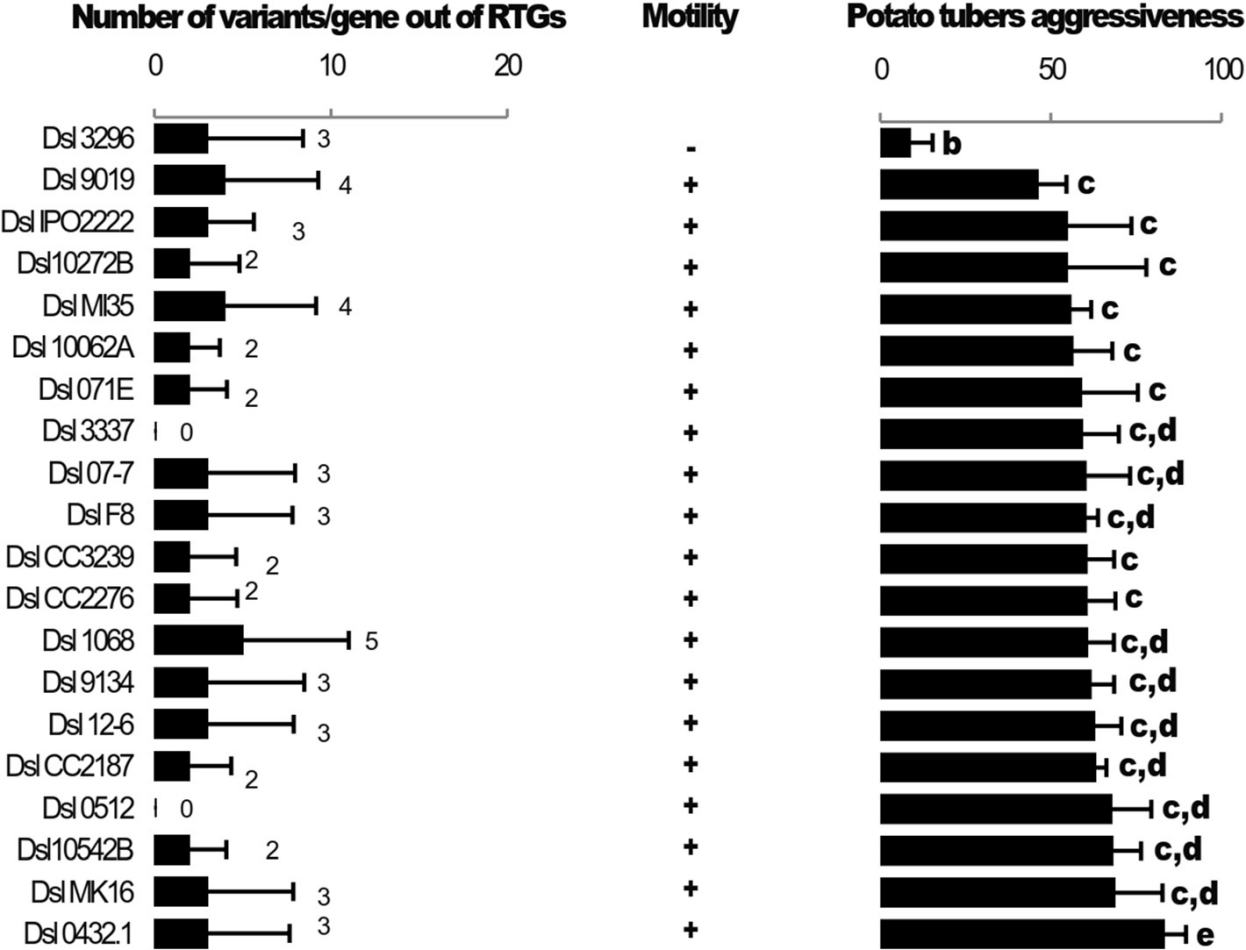
Motility and aggressiveness assays performed on potato tubers. The average of variants per gene was calculated for each strain (the RGT regions of the strain Dsl 9019, 9134 and 07-7 were omitted for calculation). The signs + and - indicate that the strain is motile or not. The letters b, c, d and e indicate statistical significance at p < 0.05 (Kruskal-walis and Tukey tests) of the aggressiveness which was measured by infecting 30 potato tubers by each of the Dsl strains

All the non-synonymous variations of the core-population were listed in Additional file 1: Table S4. Remarkably, two unique non-synonymous variations that affected the *fliC* and *fliN* flagellar genes were present in Dsl 3296. The substitution C to T at the position 952985 lead to conversion of Ala207 to Thr in FliC, while deletion of the GTC codon starting at the position 966 038 provoke the loss of the Val112 in FliN. The nucleotide variations were verified by Sanger sequencing. These two variations were unique among the sequenced *D. solani* strains, as well as the known *Dickeya* genomes (Additional file 7: Figure S6). These genes retained our attention as *fli* genes are required for aggressiveness in *Dickeya* and in other *Enterobaceteriaceace* [26–29]. We hypothesized that Dsl 3296 could be impaired in motility, hence also exhibited a reduced aggressiveness on potato host plants. We compared motility and virulence of all the 20 Dsl analyzed in this study (Fig. 8). All strains except Dsl 3296 were motile. Moreover, a weak aggressiveness of the strain 3296 was observed in virulence assay on potato tuber, hence correlating genomic variants in *fli* genes with motility and virulence deficiency. As a consequence, even if SNP/InDel variations are scarce, some of them may affect virulence functions in Dsl strains.

## Discussion

This work provided new insights into the analysis of the emerging plant-pathogen *D. solani*. We combined Illumina and PacBio technologies to determine a high quality genome sequence of *D. solani* 3337 that we used as a reference to compare 19 other genome sequences generated by Illumina technology. While previous studies reported pairwise comparison between a single *D. solani* genome with that of other *Dickeya* and *Pectobacterium* species [13, 14], this work was also based on a population genomic approach. This approach revealed the unexpected diversity of the *D. solani* genomes that resulted from a combination of scattered SNP/InDel variations as well as replacing and additive HGT events.

The majority of analyzed *D. solani* strains (16 among 20) that we called the core-population contained only 43 to 85 variants (SNPs and InDels). This result is in accordance with the high ANI values (>99.9 %) that were calculated between each strain against the reference 3337. Other studies have pointed the high homogeneity within genetic equipment of *D. solani* population [9, 12, 14, 30]. All these molecular analyses support the clonal hypothesis of the *D. solani* population. In spite of this high homogeneity, Dsl strains may exhibit some variability in aggressiveness on potato tubers [7]. A previous pairwise comparative study of two Dsl strains did not succeed in the identification of the genes and functions that could explain the different aggressiveness trait [7]. However, using a population comparative approach, we pointed out that genetic and functional variations in the motility trait could contribute to an aggressiveness decrease. This observation exemplifies the powerfulness of genomic diversity analyses on field isolates for the identification of genes that modulate aggressiveness.

Another important result was the characterization of a sub-group within the *D. solani* species, highlighting that *D. solani* population structure was more complex than described previously. The prototypic strain of this sub-group was Dsl 0512 (RNS 05.1.2A) that has been isolated from potato plant showing blackleg and soft rot symptoms in France (in 2005). The existence of the 0512 sub-group was supported by ANI value, MLSA, genomic architecture (presence of specific regions) and SNP/InDel abundance and distribution. The Dsl 0512 genome appeared as a mosaic of genes with a phylogenetic position inferred to either the *D. solani* core population or the Dsl 0512 sub-group. Remarkably, genes that belong to the Dsl 0512 phylogenetic sub-cluster have also been discovered in the 18 RGTs (143 genes) of the strain Dsl 07–7 that was also isolated in France. The involvement of the 0512 sub-group as a gene resource in replacing HGT reinforced its importance in the generation of variability in *D. solani* isolates. The strains Dsl 0512 and 07-7 showed aggressiveness level similar to that of most of the studied *D. solani* strains, suggesting that the 0512 sub-group is not associated to any particular aggressiveness behavior, at least on potato tubers.

This study also highlighted that additive and replacing HGT occurred in inter-species exchanges. Additive HGT was observed in the strain 9019 which acquired a plasmid from *B. ambifaria* AMMD. *B. ambifaria* AMMD was isolated from the rhizosphere of healthy pea plants in Wisconsin (USA) in 1985 [31] and it has been reported as very effective in controlling phytopathogenic *Pythium* species [32]. Moreover we discovered replacing HGT events that recruited *D. dianthicola* genes in the two strains Dsl 9019 (63 genes distributed in 12 RGT regions) and 9134 (39 genes in 6 RGT regions) isolated from ornamental plants (respectively Muscari and Hyacynth). These exchange events between *D. solani* and *D. dianthicola* suggested that these two pathogens could coexist in the same ecological niche. In the case of *Pectobacterium spp.*, multiple species isolations from the same infected plants have been reported [33, 34]*.* Importantly, the replacing HGT events did not correlate with an aggressiveness increase in Dsl 9019 and 9134 at least in potato tubers. However, replacing HGT generates major impact on phylogenetic inference by generating incongruities that could impair pathogen molecular diagnostics which are based on housekeeping genes. Importantly, it has been reported that succeeded HGT events between distantly related bacteria mostly implicate housekeeping genes that are also the most conserved between different species [35, 36]. Our work revealed that in the Dsl 9019 strain, the *dnaJ* and *dnaK* genes, which are usually used in phylogenetic classifications [25, 37], have been recruited from *D. dianthicola*. Since the *Dickeya* pathogens are genetically very close (ANI ≥ 93 %), replacing HGT could be predicted to interfere recurrently with taxonomical diagnostics, hence provoking assignation errors. The impact of HGT on taxonomy has been discussed in different *Enterobacteriaceae* [38, 39]. An immediate applied recommendation from our work is that even though *D. solani* is mainly described as a homogeneous population, the existence of HGT events should encourage the use of multiple taxonomical markers.

## Conclusions

As a conclusion, this work revealed that *D. solani* genomic variability may be caused by SNPs/InDels as well as replacing and additive HGT events, including plasmid acquisition. From this work, the question arises about the dynamics of the *D. solani* diversity in the course of its emergence and spreading in crop cultures. This might be further investigated by a larger scale sampling and genomic analysis.

## Methods

### Bacterial strains and growth conditions

*D. solani* strains were collected from different geographical locations and dates of isolation and also from different hosts or environments (Additional file 1: Table S1). All the strains were routinely cultured in TY medium (tryptone 5 g/L, yeast extract 3 g/L and agar 1.5 %) at 28 °C.

### DNA extraction and sequencing

Genomic DNA from each strain was extracted from overnight culture using a phenol-chloroform purification method followed by an ethanol precipitation as described by [40] Wilson. Quantity and quality control of the DNA was completed using a NanoDrop (ND 1000) device and agarose gel electrophoresis at 1.0 %.

Paired-end libraries with an insert size of 270 to 390 bp were constructed for each strain, and DNA sequencing was performed by Illumina HiSeq 2000 v3 technology. Sequencing of the library was carried out using 2×100 or 2×150 bp paired-end read module. Illumina sequencing was performed at the CNRS IMAGIF platform (Gif-sur-Yvette).

The genomic DNA of Dsl 3337 was subjected to PacBio RSII sequencing technology (Pacific Biosciences, CA, USA) using library targeted at 10kbp in insert size. Prior to assembly, short reads that are less than 500 bp were filtered off and minimum polymerase read quality used for mapping of subreads from a single zero-mode waveguides (ZMW) was set at 0.75. The 112 228 filtered reads (N50 value was 13 159 bp and total bp number was 814 445 948) were assembled using RS_HGAP_Assembly (version 3.0), which is an analysis pipeline module from Pacific Biosciences SMRT portal incorporating Celera Assembler, BLASR mapper and Quiver consensus caller algorithm. The cut-off length of seeding reads was set at 3 606 bp in order to serve as a reference for the recruitment of shorter reads for preassembly. The resulted consensus accuracy based on multiple sequence alignment of the subreads was at 99.99 %.

### Assembly, variants calling and genome sequence analysis

Assembly of the sequences was performed using the CLC Genomics Workbench v7.0.0 software (CLC Inc, Aarhus, Denmark). After quality (quality score threshold 0.05) and length (above 40 nucleotides) trimming of the sequences, contigs were generated by *de novo* assembly (CLC parameters: automatic determination of the word and bubble sizes with no scaffolding) for each strain.

Paired end reads for each strain were mapped against the reference sequence of the strain *D. solani* 3337 at mild stringency threshold (0.8 of identity on 0.5 of read length) using CLC Genomics Workbench version 7.0.0 software. The unmapped reads for each strain were collected. The mappings were used for detection of variations (SNPs and InDels) using basic variant calling tool from CLC genomic workbench version 7.0.0. Draft genome sequences composed of the contigs of each strain were used to search and analyze the variations detected. Variations with an occurrence below 99 % in the mapping step were discarded from the study.

The nucleotide identity (ANI) values were calculated as previously proposed [41] using the ANI calculator from the Kostas lab with default settings (http://enve-omics.ce.gatech.edu/ani/). Phylogenetic and molecular evolutionary analyses were conducted using MEGA, version 6 [23]. An MLSA (Multi-locus sequence analysis) was performed using eleven housekeeping genes (*rpoD, gyrB, recA, rpoS, dnaX, dnaA, gapA, fusA, rplB, purA, gyrA*) retrieved from the twenty *D. solani* strains in order to confirm their phylogenetic position within known pectinolytic *Dickeya* and *Pectobacterium* strains.

### Nucleotide sequence accession number

Draft genome sequences of *Dickeya solani* strains 9109, 0512, 9134, 07-7 have been deposited at DDBJ/EMBL/GenBank under the following accession numbers: (JWLS00000000) *D. solani* 9019, (JWMJ00000000) *D. solani* 0512, (JWLT00000000) *D. solani* 9134, (JWLR00000000) *D. solani* 07-7. The versions described in this paper are versions (JWLS01000000) *D. solani* 9019, (JWMJ01000000) *D. solani* 0512, (JWLT01000000) *D. solani* 9134, (JWLR01000000) *D. solani* 07-7. Genomes of other *Dickeya* and *Pectobacterium* species were collected from public database (Table S4).

### Aggressiveness and motility assays

Motility assays were conducted on semi-solid SM medium (beef extract at 3 g/L, peptone at 5 g/L, and 25 ml/L of 20 % glucose) with 0.5 % of agar. Two μL of an overnight bacterial suspension of each strain were used to inoculate agar plates which were incubated 16 h at 28 °C. The experiment was performed twice with 2 replicates each time.

Assessment of the aggressiveness of the strains was performed on potato tubers (cv. Binjte). To this end, 10^6^ CFU were used to infect 10 potato tubers for each strain. After 24 h of incubation at 25 °C, five aggressiveness categories were considered and attributed to tuber samples to assess the virulence of the strains. The experiments were performed three times, hence 600 tubers were infected and analyzed. The results were represented as normalized values.

Virulence assays were statistically analyzed to infer the aggressiveness variability within strains on potato tubers. Heterogeneity of strains was assessed using a Kruskal-Walis test with p < 0.05. Statistical significance of the pairwise comparisons between strains was calculated using a post hoc Tukey test with p < 0.05.

### Availability of supporting data

The alignments and phylogenetical tree for MLSA are available through the Dryad data repository doi: 10.5061/dryad.h26hs.

## Additional files

Additional file 1: Tables S1-S5.
**Table S1.** *Dickeya solani* strains in this study. **Table S2.** Sequencing data and mappings on the Dsl 3337 genome. **Table S3** Variants distribution on the strains *vs.* Dsl 3337. **Table S4.** Non-synonymous variants: this table shows the unique and the shared variants within homoge:nous *D. solani* strains (MK16, MIE35, 0432.1, 12-6, F8, 1068, 3296, 07E, 10062A, 10272B, 10542B, 2187, 2276, 3239, IPO2222^T^). **Table S5.** Other genomes used in this study (DOC 130 kb) Additional file 2: Figure S1. Synteny between the strain *D. solani* 3337 and the draft genome *D. solani* 0512. The alignment was performed using MAUVE software, underlining a high conservation of the synteny. The numbers indicate the positions of the strain-specific genomic regions generated by *de novo* assembly of the unmapped reads. (TIFF 956 kb) Additional file 3: Figure S2. Protein-based phylogenetic trees revealing Dsl 0512 as a member of in distinct sub-cluster within the *D. solani* species. (TIFF 2843 kb) Additional file 4: Figure S3. Protein-based phylogenetic trees of different RGTs in Dsl 07-7. The genes were retrieved from RGT1, RGT2, RGT3, RGT6, RGT10 and RGT12 of Dsl 07-7. The phylogenetic positions indicate replacing HGT events from the *D. solani* 0512 sub-group. (TIFF 2881 kb) Additional file 5: Figure S4. Protein-based phylogenetic trees of different RGTs in Dsl 9134. The genes were retrieved from RGT1, RGT3, RGT5 and RGT6 of Dsl 9134. The phylogenetic positions highlight replacing HGT events from the *D. dadantii* species. (TIFF 2117 kb) Additional file 6: Figure S5. Protein-based phylogenetic trees of different RGTs in Dsl 9019. The genes were retrieved from RGT1, RGT2, RGT3, RGT4, RGT5 and RGT10 of Dsl9019. The phylogenetic positions highlight replacing HGT events from the *D. dadantii* species. (TIFF 2989 kb) Additional file 7: Figure S6. Local alignment of FliC and FliN proteins. The variations at the positions 207 in FliC and 112 in FliN are indicated in red color, other variations are in blue color. Amino acid position is numbered according to the *D. solani* 3337 sequence of FliC and FliN. We used the draft and complete genomes of the 19 *D. solani* sequenced in this study, those of *D. solani* strains GBCC2040 and MK10, 15 *D. dianthicola* including the strains MIE32, MIE33, MIE34, CFBP1888, CFBP2015, CFBP2982, RNS04.9, RNS10.20.2A, RNS11.47.1A, DW04.9 K, DS05.3.3, GBBC2039, IPO980, NBPPB3534 and NCPPB453, *D. dadantii* strains 3937, NCPPB898 and NCPPB3537, *D. chrysanthemi* strains NCPPB3533 and NCPPB516, and *D. zeae* strains Ech1591 and NCPPB2538. (TIFF 1283 kb)

## Abbreviations

ANI: Average nucleotide identity
CDS: Coding DNA sequence
Dsl: *Dickeya solani*
HGT: Horizontal gene transfer
InDel: Insertion deletion
MLSA: Multi-locus sequence analysis
NCBI: National center for biotechnology information
RGT: Replacing HGT
SNP: Single nucleotide polymorphism
