# Supplementary material for: Population genomics reveals additive and replacing horizontal gene transfers in the emerging pathogen Dickeya solani
Source: BMC Genomics. 2015 Oct 14;16:788. doi: 10.1186/s12864-015-1997-z (PMC4607151; doi:10.1186/s12864-015-1997-z)
Supplement: Additional file 1: Tables S1-S5. — Table S1. Dickeya solani strains in this study. Table S2. Sequencing data and mappings on the Dsl 3337 genome. Table S3 Variants distribution on the strains vs. Dsl 3337. Table S4. Non-synonymous variants: this table shows the unique and the shared variants within homoge:nous D. solani strains (MK16, MIE35, 0432.1, 12-6, F8, 1068, 3296, 07E, 10062A, 10272B, 10542B, 2187, 2276, 3239, IPO2222T). Table S5. Other genomes used in this study (DOC 130 kb) [file 12864_2015_1997_MOESM1_ESM.doc]

Table S1 *Dickeya solani* strains in this study

| **Organisms** | **Code** | **Host&Environment** | **Geography** | **Year** |
| --- | --- | --- | --- | --- |
| *D. solani* PPO 9019 | Dsl 9019 | Muscari | Netherlands | 2006 |
| *D. solani* PPO 9134 | Dsl 9134 | Hyacynth | Netherlands | 2008 |
| *D. solani* RNS 05.1.2A | Dsl 0512 | Potato | France | 2005 |
| *D. solani* RNS 07.7.3B | Dsl 07-7 | Potato | France | 2007 |
| *D. solani* RNS 08.23.3.1A | Dsl 3337 | Potato | France | 2008 |
| *D. solani* RNS 10.68.1A | Dsl 1068 | Potato | France | 2010 |
| *D. solani* RNS 12.6.1A | Dsl 12-6 | Potato | Switzerland | 2012 |
| *D. solani* EU3296 (=GRC77) | Dsl 3296 | Potato | Israël | 2008 |
| *D. solani* F8 (=PPL0433) | Dsl F8 | Potato | Finland | 2004 |
| *D. solani* RNS 06/026 | Dsl MIE35 | Potato | Switzerland | 2005 |
| *D. solani* Ds0432.1 | Dsl 0432.1 | Potato | Finland | 2004 |
| *D. solani* MK16 | Dsl MK16 | River water | Scotland | ND |
| *D. solani* 07-1E | Dsl 07E | Potato | France | 2007 |
| *D. solani* 10-06-2A | Dsl10062A | Potato | France | 2010 |
| *D. solani* 10-27-2A | Dsl10272A | Potato | Switzerland | 2010 |
| *D. solani* 10-54-2B | Dsl10542B | Potato | France | 2010 |
| *D. solani* CC2187 | Dsl 2187 | Potato | Ireland | - |
| *D. solani* CC2276 | Dsl 2276 | Potato | Poland | - |
| *D. solani* CC3239 | Dsl 3239 | Potato | United Kingdom | - |
| *D. solani* IPO2222 ^T^ | Dsl IPO2222 | Potato | Netherlands | 2007 |

Table S2 Sequencing data and mappings on the Dsl 3337 genome

| **Strain** | **Total reads** | **Mapped reads (%)** | **Unmapped reads(%)** | **mean covrage** |
| --- | --- | --- | --- | --- |
| Dsl0512 | 37 334 298 | 92.34 | 7.66 | 700 |
| Dsl07-7 | 41 013 624 | 99.97 | 0.03 | 800 |
| Dsl9019 | 48 631 564 | 98.08 | 1.92 | 900 |
| Dsl9134 | 41 625 672 | 99.53 | 0.47 | 800 |
| Dsl3296 | 40 769 110 | 99.45 | 0.55 | 800 |
| DslF8 | 46 613 616 | 99.58 | 0.42 | 900 |
| Dsl12-6 | 44 112 694 | 99.84 | 0.16 | 850 |
| Dsl0432.1 | 23 156 694 | 99.58 | 0.42 | 450 |
| Dsl1068 | 38 735 222 | 99.98 | 0.02 | 750 |
| DslMK16 | 19 253 116 | 99.62 | 0.38 | 350 |
| DslMI35 | 18 400 330 | 99.59 | 0.41 | 350 |
| Dsl 07E | 11 857 158 | 99.28 | 0.72 | 400 |
| Dsl10062A | 12 928 577 | 99.35 | 0.65 | 440 |
| Dsl10272B | 11 889 376 | 99.41 | 0.59 | 400 |
| Dsl10542B | 15 319 899 | 99.90 | 0.10 | 520 |
| Dsl 2187 | 14 653 859 | 99.15 | 0.85 | 500 |
| Dsl 2276 | 17 475 360 | 99.54 | 0.46 | 590 |
| Dsl 3239 | 17 475 360 | 99.37 | 0.63 | 516 |
| Dsl IPO2222 | 16 403 141 | 99.90 | 0.10 | 543 |

Table S3 Variants distribution on the strains *vs.* Dsl 3337

| **Strains** | **Variants count** | **Variants out CDS** | **Variants in CDS** | **Non synonymous variants** |
| --- | --- | --- | --- | --- |
| Dsl0512 | 37493 | 5350 | 32143 | 7234 |
| Dsl07-7 | 1454 | 146 | 1308 | 292 |
| Dsl9019 | 3433 | 331 | 3102 | 566 |
| Dsl9134 | 2815 | 289 | 2526 | 542 |
| Dsl3296 | 85 | 19 | 66 | 18 |
| DslF8 | 65 | 18 | 47 | 13 |
| Dsl12-6 | 63 | 20 | 43 | 12 |
| Dsl0432.1 | 76 | 18 | 58 | 11 |
| Dsl1068 | 70 | 18 | 52 | 13 |
| DslMK16 | 62 | 19 | 43 | 12 |
| DslMI35 | 75 | 19 | 56 | 14 |
| Dsl 07E | 49 | 14 | 35 | 6 |
| Dsl10062A | 48 | 14 | 34 | 10 |
| Dsl10272B | 43 | 13 | 30 | 6 |
| Dsl10542B | 45 | 12 | 33 | 8 |
| Dsl 2187 | 51 | 16 | 35 | 8 |
| Dsl 2276 | 44 | 13 | 31 | 8 |
| Dsl 3239 | 52 | 16 | 36 | 10 |
| Dsl IPO2222 | 49 | 15 | 34 | 8 |

Table S4 Non-synonymous variants: this table shows the unique and the shared variants within homoge:nous *D. solani* strains (MK16, MIE35, 0432.1, 12-6, F8, 1068, 3296, 07E, 10062A, 10272B, 10542B, 2187, 2276, 3239, IPO2222).

| **Position** | **CDS affected** | **Number of strains** |
| --- | --- | --- |
| 1315848 | Aconitate hydratase 2 | 8 |
| 1315885 | Aconitate hydratase 2 | 15 |
| 1316262 | Aconitate hydratase 2 | 15 |
| 1466994 | Putative sensory histidine kinase YfhA | 2 |
| 1471936 | Phosphoribosylformylglycinamidine synthase | 1(DslCC2276) |
| 1598278 | Putative sugar ABC transport system | 2 |
| 1704637 | RNA polymerase sigma factor RpoS | 1(Dsl3296) |
| 2492465 | hypothetical protein | 7 |
| 2492472 | hypothetical protein | 10 |
| 2495908 | Exo-poly-alpha-D-galacturonosidase precursor | 14 |
| 2496505 | Exo-poly-alpha-D-galacturonosidase precursor | 7 |
| 2754775 | rRNA small subunit 7-methylguanosine (m7G) | 1(DslMK16) |
| 2930935 | VfmB protein | 2 |
| 3776452 | hypothetical protein | 1(DslMIE35) |
| 3797586 | putative RTX toxin | 11 |
| 3802848 | putative RTX toxin | 7 |
| 3803181 | putative RTX toxin | 7 |
| 3803457 | putative RTX toxin | 8 |
| 4215221 | Cytochrome d ubiquinol oxidase subunit I | 1(DslIPO2222) |
| 4325693 | VgrG protein | 15 |
| 441030 | Putative large exoprotein | 1(DslCC3239) |
| 441084 | Putative large exoprotein | 1(DslCC3239) |
| 441285 | Putative large exoprotein | 1(DslCC3239) |
| 4660124 | Malonate decarboxylase alpha subunit | 1(DslCC2187) |
| 4889553 | Fructose-specific phosphocarrier protein HPr | 1(Dsl10542B) |
| 4917738 | Methyl-accepting chemotaxis protein I | 3 |
| 4917893 | Methyl-accepting chemotaxis protein I | 3 |
| 4918002 | Methyl-accepting chemotaxis protein I | 1(DslCC2276) |
| 521861 | Tripeptide aminopeptidase | 1(DslMK16) |
| 952985 | Flagellar biosynthesis protein FliC | 1(Dsl3296) |
| 966036 | Flagellar motor switch protein FliN | 1(Dsl3296) |

Table S5 Other genomes used in this study

| **Organisms** | **Accession n°.** | **Code** |
| --- | --- | --- |
| *Dickeya dianthicola* NCPPB 453^T^ | | [CM001841.1](http://www.ncbi.nlm.nih.gov/nuccore/CM001841.1) |  | | --- | --- | | Ddi 453^T^ |
| *Dickeya dianthicola* NCPPB 2039 | | [CM001838.1](http://www.ncbi.nlm.nih.gov/nuccore/CM001838.1) |  | | --- | --- | | Ddi2039 |
| *Dickeya dianthicola* RNS 04.9 | Unpublished | Ddi RNS 04.9 |
| *Dickeya chrysanthemi* NCPPB 516 | | [CM001904.1](http://www.ncbi.nlm.nih.gov/nuccore/CM001904.1) |  | | --- | --- | | Dch 516 |
| *Dickeya chrysanthemi* NCPPB3533 | |  | [CM001981.1](http://www.ncbi.nlm.nih.gov/nuccore/CM001981.1) | | --- | --- | | Dch 3533 |
| *Dickeya zeae* NCPPB 3532 | [CM001858.1](http://www.ncbi.nlm.nih.gov/nuccore/CM001858.1) | Dz 3532 |
| *Dickeya zeae* NCPPB2538 | CM001977.1 | Dz 2538 |
| *Dickeya dadantii* 3937 | | [CP002038.1](http://www.ncbi.nlm.nih.gov/nuccore/CP002038.1) |  | | --- | --- | | Dda 3937 |
| *Dickeya dadantii* NCPPB 898^T^ | | [CM001976.1](http://www.ncbi.nlm.nih.gov/nuccore/CM001976.1) |  | | --- | --- | | Dda898^T^ |
| *Pectobacterium atrosepticucm* SCRI1043 | | [BX950851.1](http://www.ncbi.nlm.nih.gov/nuccore/BX950851.1) |  | | --- | --- | | Pa SCRI1043 |
| *Pectobacterium carotovorum* susbp. *carotovorum* PC1 | |  | [CP001657.1](http://www.ncbi.nlm.nih.gov/nuccore/CP001657.1) |  | | --- | --- | --- | | Pcc PC1 |
