# Supplementary figures and images for: Population genomics reveals additive and replacing horizontal gene transfers in the emerging pathogen Dickeya solani
Source: BMC Genomics. 2015 Oct 14;16:788. doi: 10.1186/s12864-015-1997-z (PMC4607151; doi:10.1186/s12864-015-1997-z)

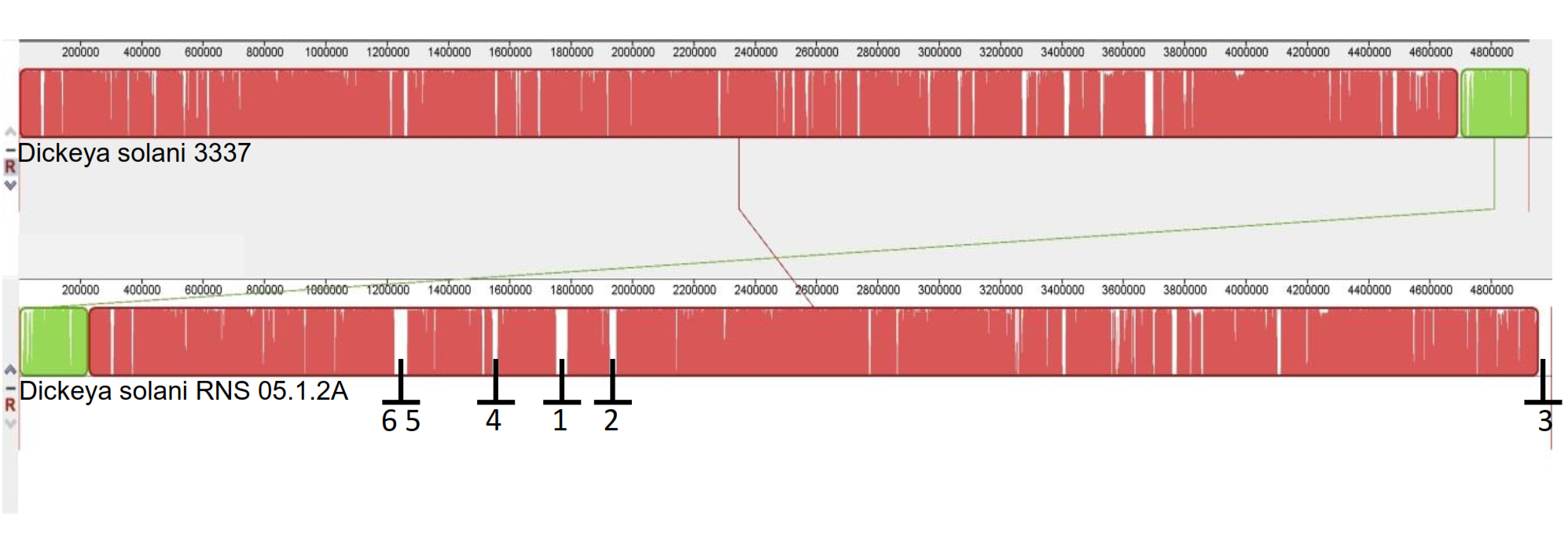

Supplement: Additional file 2: Figure S1. — Synteny between the strain D. solani 3337 and the draft genome D. solani 0512. The alignment was performed using MAUVE software, underlining a high conservation of the synteny. The numbers indicate the positions of the strain-specific genomic regions generated by de novo assembly of the unmapped reads. (TIFF 956 kb) [file 12864_2015_1997_MOESM2_ESM.tif]

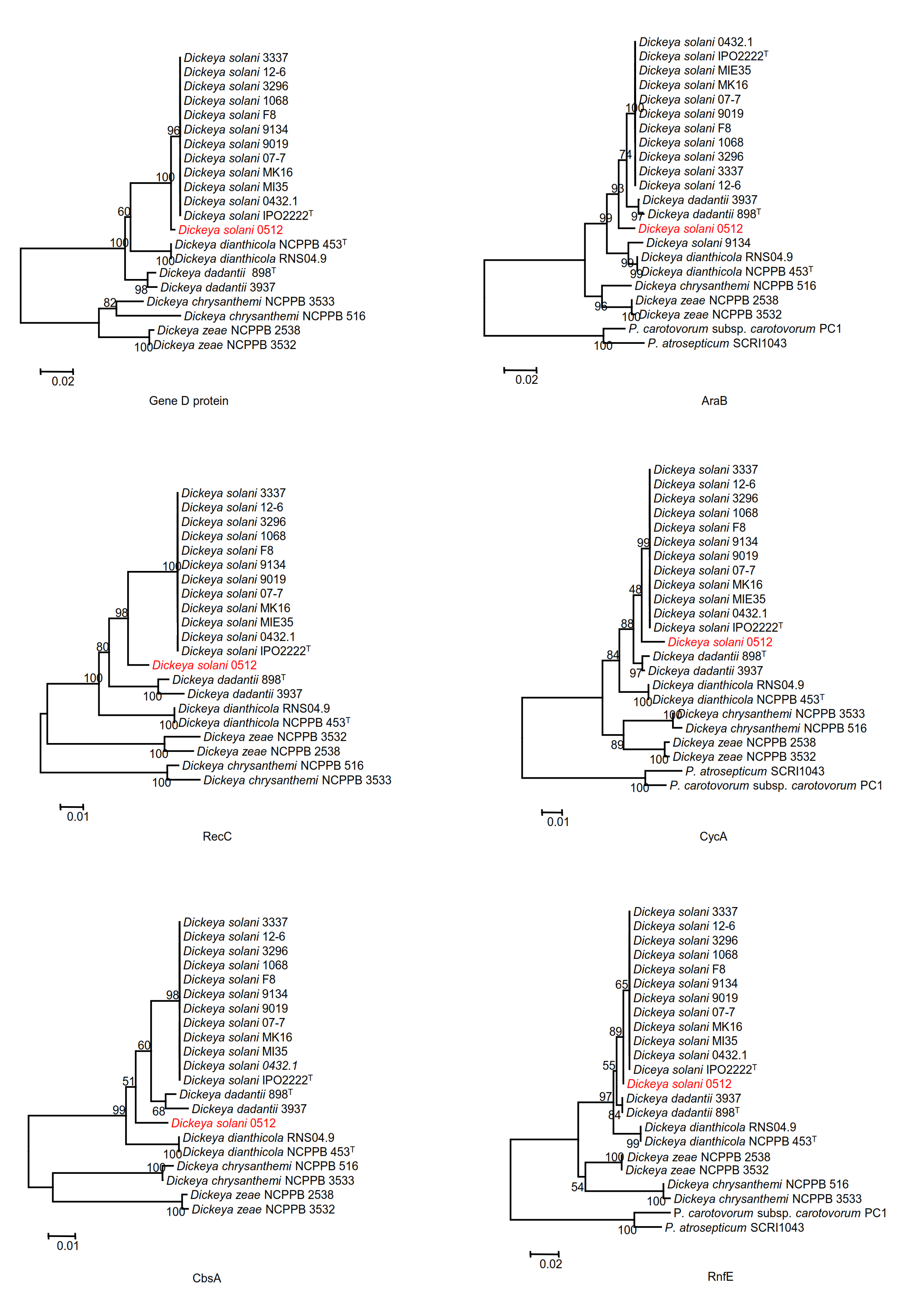

Supplement: Additional file 3: Figure S2. — Protein-based phylogenetic trees revealing Dsl 0512 as a member of in distinct sub-cluster within the D. solani species. (TIFF 2843 kb) [file 12864_2015_1997_MOESM3_ESM.tif]

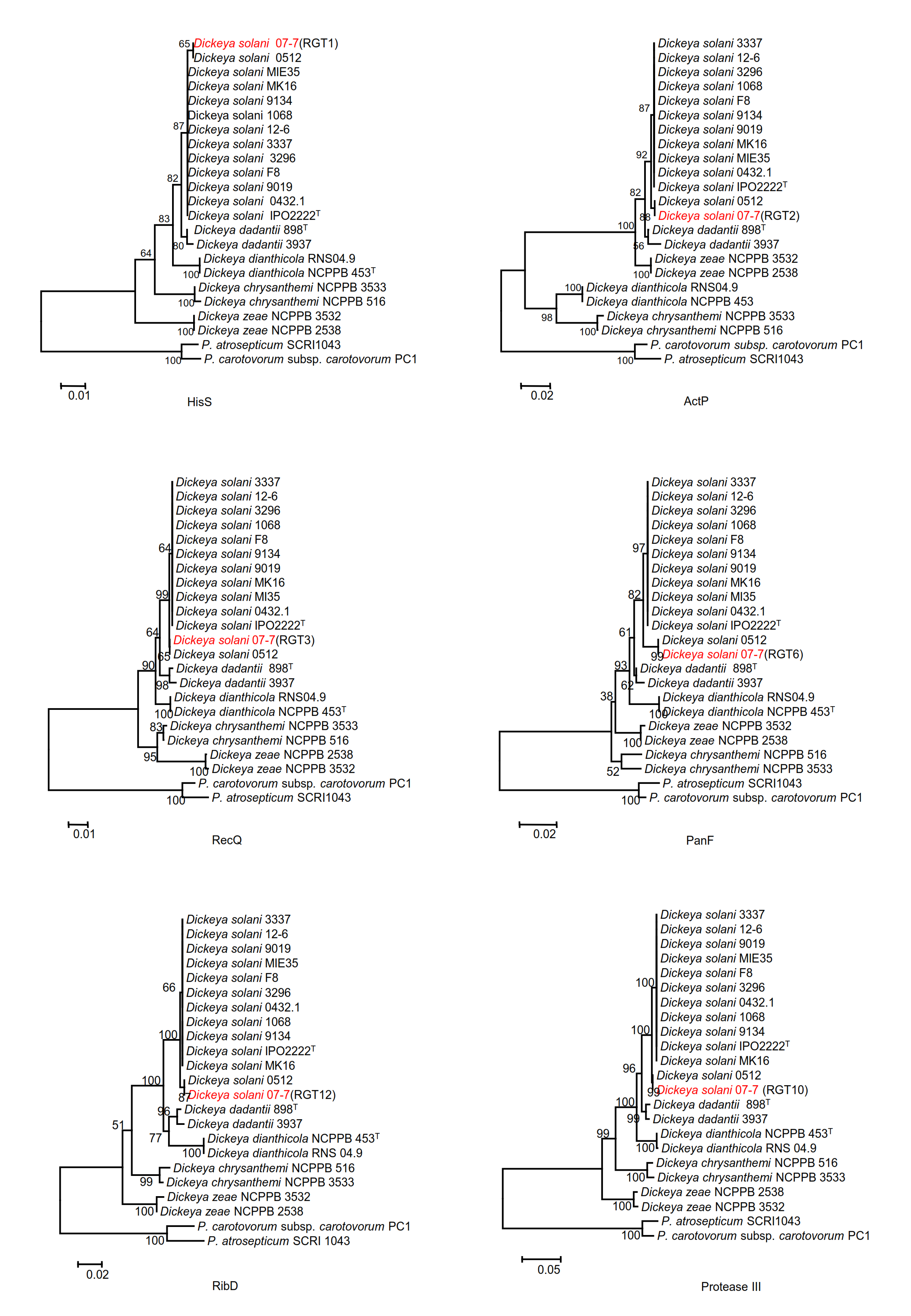

Supplement: Additional file 4: Figure S3. — Protein-based phylogenetic trees of different RGTs in Dsl 07-7. The genes were retrieved from RGT1, RGT2, RGT3, RGT6, RGT10 and RGT12 of Dsl 07-7. The phylogenetic positions indicate replacing HGT events from the D. solani 0512 sub-group. (TIFF 2881 kb) [file 12864_2015_1997_MOESM4_ESM.tif]

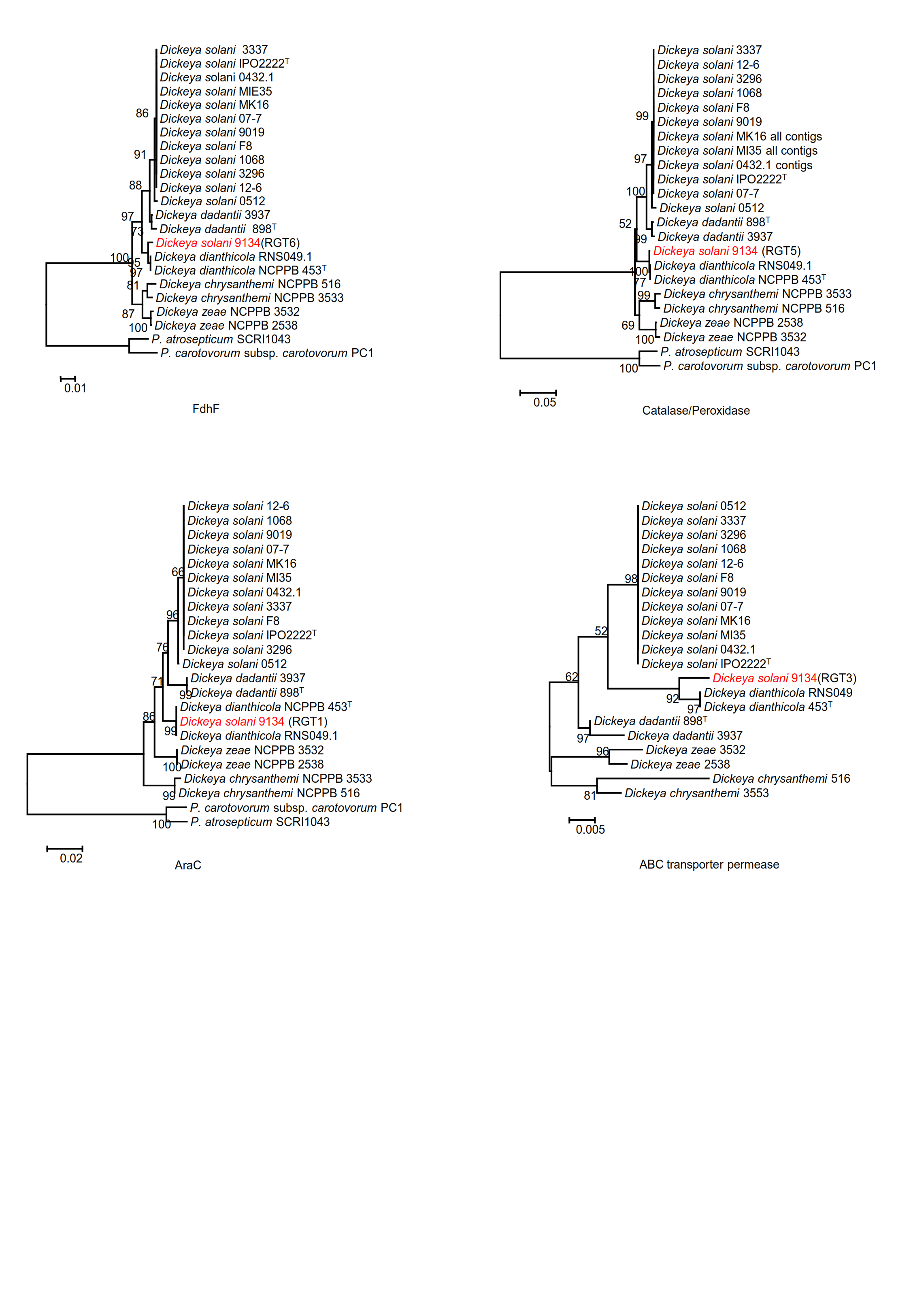

Supplement: Additional file 5: Figure S4. — Protein-based phylogenetic trees of different RGTs in Dsl 9134. The genes were retrieved from RGT1, RGT3, RGT5 and RGT6 of Dsl 9134. The phylogenetic positions highlight replacing HGT events from the D. dadantii species. (TIFF 2117 kb) [file 12864_2015_1997_MOESM5_ESM.tif]

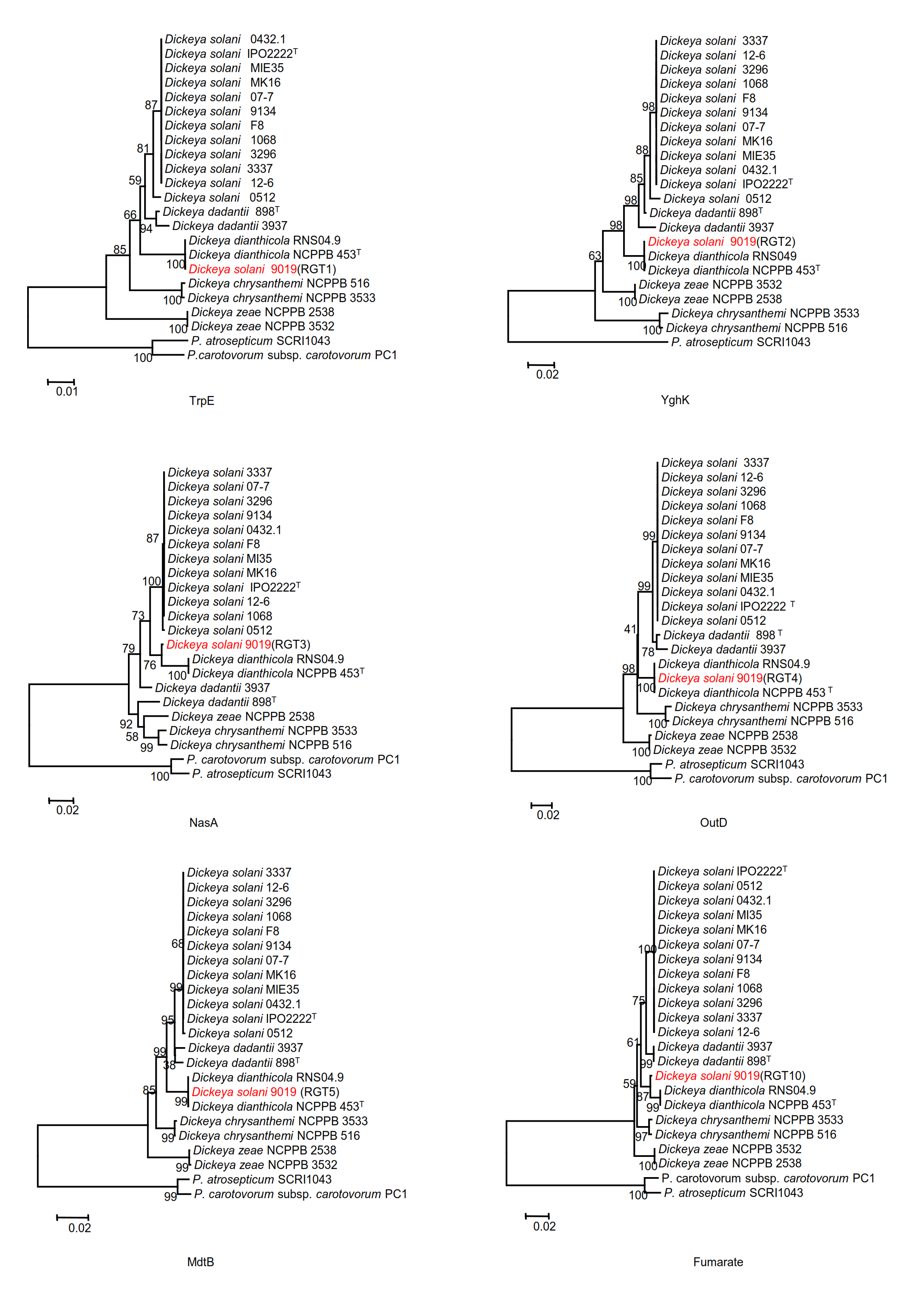

Supplement: Additional file 6: Figure S5. — Protein-based phylogenetic trees of different RGTs in Dsl 9019. The genes were retrieved from RGT1, RGT2, RGT3, RGT4, RGT5 and RGT10 of Dsl9019. The phylogenetic positions highlight replacing HGT events from the D. dadantii species. (TIFF 2989 kb) [file 12864_2015_1997_MOESM6_ESM.tif]

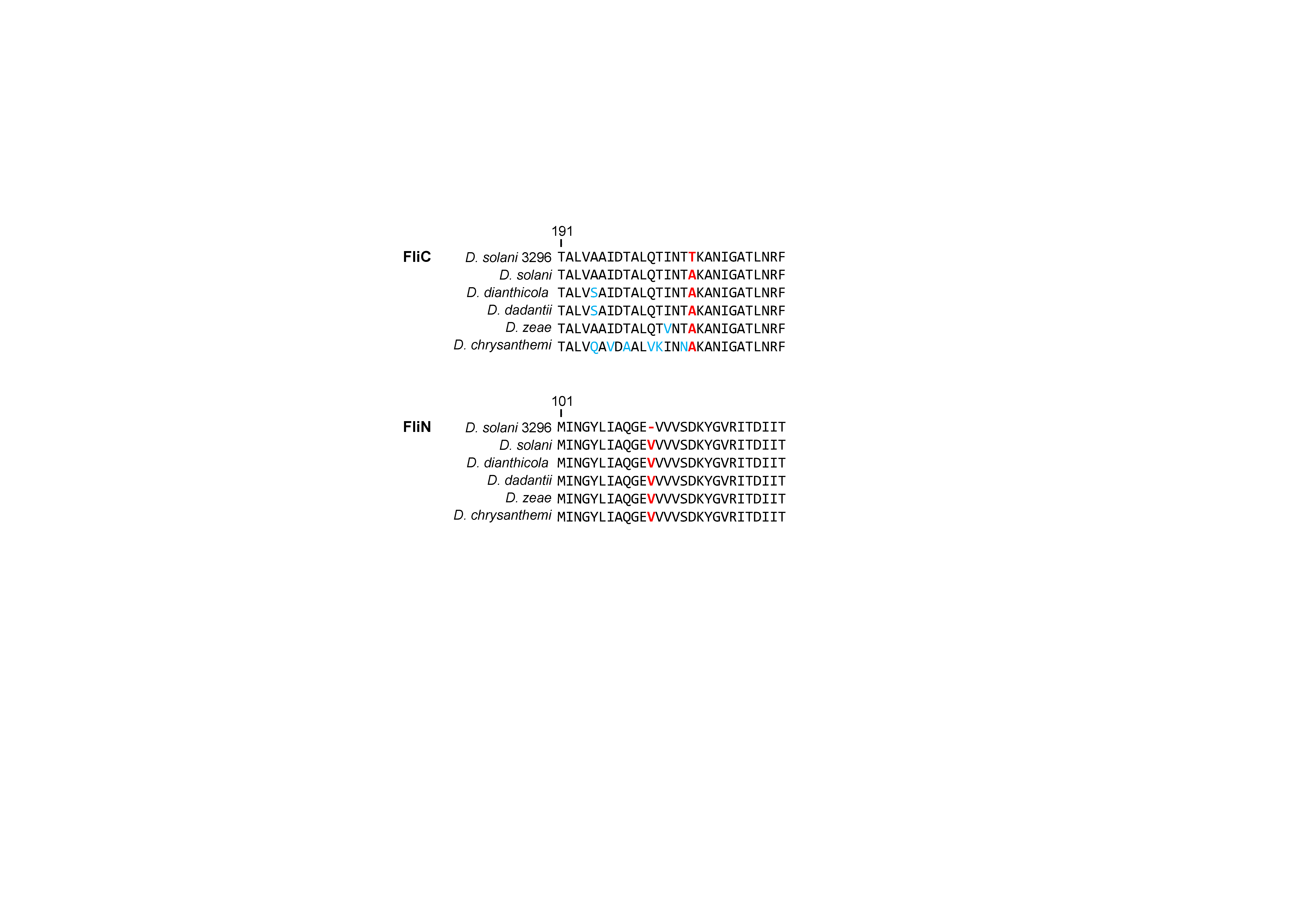

Supplement: Additional file 7: Figure S6. — Local alignment of FliC and FliN proteins. The variations at the positions 207 in FliC and 112 in FliN are indicated in red color, other variations are in blue color. Amino acid position is numbered according to the D. solani 3337 sequence of FliC and FliN. We used the draft and complete genomes of the 19 D. solani sequenced in this study, those of D. solani strains GBCC2040 and MK10, 15 D. dianthicola including the strains MIE32, MIE33, MIE34, CFBP1888, CFBP2015, CFBP2982, RNS04.9, RNS10.20.2A, RNS11.47.1A, DW04.9 K, DS05.3.3, GBBC2039, IPO980, NBPPB3534 and NCPPB453, D. dadantii strains 3937, NCPPB898 and NCPPB3537, D. chrysanthemi strains NCPPB3533 and NCPPB516, and D. zeae strains Ech1591 and NCPPB2538. (TIFF 1283 kb) [file 12864_2015_1997_MOESM7_ESM.tiff]
